# Minimally invasive surgery for intra-articular calcaneus fractures: a 9-year, single-center, retrospective study of a standardized technique using a 2-point distractor

**DOI:** 10.1186/s12891-020-03762-9

**Published:** 2020-11-14

**Authors:** Christian Rodemund, Ronny Krenn, Carl Kihm, Iris Leister, Reinhold Ortmaier, Werner Litzlbauer, Angelika M. Schwarz, Georg Mattiassich

**Affiliations:** 1 AUVA - Traumacenter (UKH) Linz, Teaching Hospital of the Paracelsus Medical University Salzburg, Linz, Austria; 2 Norton Audubon Hospital, Attending Podiatric Surgeon, Louisville, KY USA; 3 Institute of Molecular Regenerative Medicine, Paracelsus Medical University, Salzburg, Austria; 4 Department of Orthopaedic Surgery, Ordensklinikum Barmherzige Schwestern Linz, Vinzenzgruppe Center of Orthopaedic Excellence, Teaching Hospital of the Paracelsus Medical University Salzburg, Linz, Austria; 5 Research Unit of Orthopedic Sports Medicine and Injury Prevention, Institute for Sports Medicine, Alpine Medicine and Health Tourism (ISAG), UMIT, Hall in Tirol, Austria; 6 AUVA - Traumacenter (UKH) Styria | Graz, Teaching Hospital of the Medical University Graz, Göstinger Straße 24, 8020 Graz, Austria

**Keywords:** Calcaneal fracture, Minimally invasive, Intra-articular fracture

## Abstract

**Background:**

A fracture of the calcaneus can be a painful and disabling injury. Treatment modalities may be conservative or operative. Surgical treatment strategies include open reduction and internal fixation (ORIF) techniques, as well as a variety of minimally invasive methods. The aim of this study was to evaluate the treatment options and post-treatment complication rates for intra-articular calcaneal fractures at the Traumacenter Linz over a 9-year period.

**Methods:**

All patients with calcaneal fractures treated at the Traumacenter Linz between 2007 and 2015 were included in this study. The patients records were retrospectively reviewed, and the data, including demographic parameters, cause of injury, and the time between injury and operative treatment were analyzed. The number of secondary operative interventions due to soft-tissue complications, hardware removal, and the long-term arthrodesis rate were evaluated.

**Results:**

A minimally invasive 2-point-distractor method was used in 85.8% (*n* = 182) of all operatively managed calcaneal fractures (*n* = 212) in our department. The majority of the operations (88.7%) were performed within 2 days after the accident. The secondary operation rate resulting from wound complications was 2.7% in the 2-point distractor group and 16.7% in the ORIF group. A secondary arthrodesis was performed in 4.7% (*n* = 9) of the subtalar joints in the entire study population.

**Conclusions:**

Our data supported the assumption that severe wound complications would be less likely to occur after minimally invasive treatment compared to ORIF treatment. The rate of secondary arthrodesis in the study cohort was comparable to that in the literature.

**Level of evidence:**

IV

**Supplementary Information:**

The online version contains supplementary material available at 10.1186/s12891-020-03762-9.

## Background

A calcaneal fracture can be a painful and disabling injury. Fractures of the heel bone account for about 2% of all fractures, and are often intra-articular, multi-fragmentary, and comminuted. Standardized treatment protocols are lacking, and the optimal treatment of intra-articular calcaneal fractures is still controversial. Multiple factors such as fracture pattern, comorbidities, timing, and status of the soft tissue must be considered [1–3].

Treatment modalities vary between conservative, open reduction and internal fixation (ORIF), numerous minimally invasive approaches, and even primary subtalar joint arthrodesis [4–6].

Prolonged and eventful healing or mal-reduction of the fracture can lead to poor results and a persistent disability [1, 3]. The goal of operative management is to retrieve an anatomically correct reduction of the joint surfaces and a reconstruction of the length, width, height, and axis of the calcaneus. However, an anatomic reconstruction cannot always be achieved in severely comminuted fractures [6, 7]. An optimal treatment should minimize operative soft tissue dissection, which reduces the risk of wound dehiscence and does not compromise potentially necessary surgical procedures in adjacent tissues [3, 8]. If a secondary arthrodesis of the subtalar joint is required later on, the procedure is generally easier to perform after a previous minimally invasive procedure. This will lead to a better clinical result since the calcaneal axis has already been corrected, and the bone stock has been remodeled [5, 9].

Accounting for the advantages of operative treatment in general, and minimally invasive treatment in particular, we implemented a treatment protocol in our department and standardized the operative technique from positioning to X-ray views, repositioning and osteosynthesis, and postoperative care.

The aim of this study was to evaluate this treatment protocol and the subsequent complication rate in the management of intra-articular calcaneal fractures over a 9-year period. Herein, we also describe our operative techniques and protocol for the management of open or closed calcaneal fractures (see Additional file 1).

## Methods

### Study design and participants

A retrospective data-analysis was performed on the data of 298 patients at the Traumacenter Linz, Austria. Between 1/1/2007 and 1/1/2016, patients with uni- or bilateral, open or closed calcaneal fractures, treated operatively or conservatively with a follow-up of at least 12 months were included in the study. Patients were analyzed using data extracted from the medical documentation system of the Austrian Social Insurance for Occupational Risks (AUVA).

Demographic data, cause of injury, time from injury to surgery, treatment modality, need for revision surgery, and complications were analyzed.

### Complications

Complications were defined as postoperative wound healing problems that required revision surgery, unplanned hardware removal due to irritation of the soft tissue, or the need for a subtalar arthrodesis. An elective hardware removal was not considered to be a complication. Elective hardware removal procedures were performed with the intention to avoid interference with a potential arthrodesis in the future or on an explicit request from the patient.

### Statistical analysis

Descriptive statistics, including means and standard deviations (SDs) for continuous variables (age, time from accident to operative intervention) and frequency counts for categorical variables (sex, treatment modality, trauma mechanism, operative technique, infectious complications, number of implant removals, cases necessitating secondary subtalar arthrodesis) were calculated. Chi-square analyses were used as appropriate to determine whether there were differences between the two surgically treated groups. All statistical analyses were performed using SPSS statistical software (version 23, SPSS Inc., Chicago, Illinois, USA), and *p* < .05 indicated a statistically significant result.

## Results

### Demographics

From 2007 to 2015, a total of 298 patients with calcaneal fractures treated at the Traumacenter Linz were identified. Of these, 236 were men (79.2%) and 62 were women (20.8%). In total, 212 patients (71.1%) were treated operatively, and 86 patients (28.9%) were treated conservatively. Patient age at the time of injury ranged from 15 to 82 years. The mean age was 45.7 (range, 15–79) years, and 44.2 (range, 7–94) years in the operatively and conservatively treated groups, respectively. Details see Table 1.

**Table 1:** Demographics of the patients with a calcaneus fracture treated at the Traumacenter Linz from 2007 to 2015. In total *n* = 298 patients were observed, 71% (*n* = 212 / 298) were treated operatively, 29% underwent conservative care. An odds ratio of 3:1 (male: female) could be interpreted in both patient groups with a balanced age distribution

Procedure	Sex	N	Age in years				
			Min	Max	Median	Mean	SD
OP	Men	174	16	79	46	45.3	13,5
	Women	38	15	73	49.5	48.0	12,5
	Total	212	15	79	46	45.7	13,3
Conservative	Men	62	15	82	42.5	44.0	18,9
	Women	24	7	94	46.5	44.7	23,7
	Total	86	7	94	44.5	44.2	20,3

*SD* Standard deviation, *OP* Operative, *Min* Minimum, *Max* Maximum

### Trauma mechanism

The most common injuries were ground-level falls (29%), occurring mostly in elderly female patients. Falls from a height of more than two meters occurred in 30% of cases, mostly in young male patients. External trauma, such as motor vehicle accidents, occurred in 33% of these patients.

### Operative technique

**Fig. 1:**
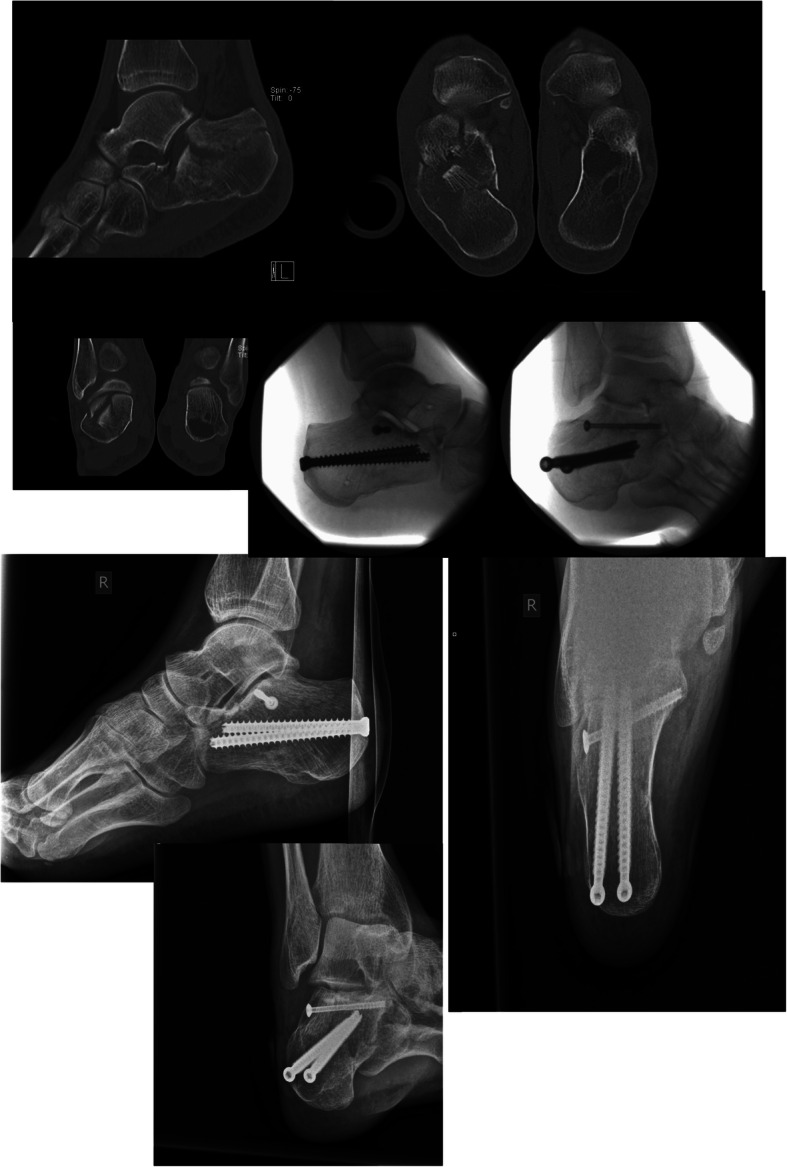
Patient case: A highly comminuted calcaneus fracture in depression-type form, treated by minimally invasive surgery. A case of a 42 years at operation-time, male patient, treated at the Traumacenter Linz in 2010

The operative technique is given to supplement this article. Within the 9-year period, 212 patients were operatively treated at the Traumacenter Linz, and the majority were treated using the 2-point distraction method (182 patients, 85.8%); a patient case is displayed in Fig. 1. Only seven patients were treated with ORIF (3.3%). K-wire fixations, or combinations of plates and K-wires with or without utilizing the 2-point distractor were performed on 23 patients (10.8%). The annual distribution of the operative techniques is presented in Table 2.

**Table 2:** Annual distribution of the operative calcaneus fractures methods at the Traumacenter Linz from 2007 to 2015. Almost the entire patient collective was treated by the minimally invasive technique with the 2-point distractor (85.8% versus 10.8%), which represents a large study collective and indicates the effectiveness of this technique

Year	ORIF		MIT with the 2-point-distractor		K-Wire fixation or another combination		Total
	N	% per year	N	% per year	N	% per year	N
2007	1	5.3%	16	84.7%	2	10.5%	19
2008	3	13.6%	17	77.3%	2	9.1%	22
2009	0	0.0%	27	90.0%	3	10.0%	30
2010	2	8.0%	19	76.0%	4	16.0%	25
2011	0	0.0%	24	96.0%	1	4.0%	25
2012	0	0.0%	20	87.0%	3	13.0%	23
2013	1	4.5%	17	77.3%	4	18.2%	22
2014	0	0.0%	24	88.9%	3	11.1%	27
2015	0	0.0%	18	94.7%	1	5.3%	19
Total	7	3.3%	182	85.8%	23	10.8%	212

*MIT* Minimally invasive technique, *ORIF* Open reduction and internal fixation, *KW* Kirschner-Wire

### Time from accident to operative intervention

The operative interventions were performed within the first 2 days after injury in 88.7% of the patients. Most patients, who underwent surgery later than 2 days post-injury (3–14 days) were transferred from other hospitals or had multiple injuries resulting in a delayed treatment of their calcaneal fractures.

### Complications

Due to the low number of patients that underwent ORIF, their data were pooled with that of the group treated with K-wires or a combination of K-wires and plates (30 patients) to compare with the minimally invasive operations group (182 patients).

In total, wound complications that required revision occurred in 4.7% (10 patients). Five out of 182 patients in the minimally invasive group (2.7%), and 5 out of 30 patients in the other two surgically treated groups (16.7%) sustained a post-operative infection (chi-square statistic = 11.1; *p* = 0.000862). Specifics see Table 3.

**Table 3:** Overview of the occurrence of complications regarding deep infections in the operative patients care. A total collective rate of 2.7% versus 16.7% could be observed, representing a low complication rate in the minimally invasive treatment. The modified technique showed that severe wound complications are less likely to occur after the minimally invasive procedure compared to ORIF or/and additional KW

Year	Deep infection - MIT		Deep infection - ORIF + KW		Total
	N	% per year	N	% per year	N
2007	1	5.3%	0	0.0%	1
2008	0	0.0%	1	4.5%	1
2009	0	0.0%	1	3.3%	1
2010	1	4.0%	1	4.0%	2
2011	1	4.0%	1	4.0%	2
2012	1	4.3%	0	0.0%	1
2013	0	0.0%	1	4.5%	1
2014	0	0.0%	0	0.0%	0
2015	1	5.3%	0	0.0%	1
Total	5	2.7%	5	16.7%	10

*MIT* Minimally invasive technique, *ORIF* Open reduction and internal fixation, *KW* Kirschner-Wire

Unplanned hardware removals due to irritation of the soft tissue or adjacent joints were performed in 4.2% of the study population. The secondary subtalar arthrodesis rate was 4.7% in the whole collective at the time of the data analysis (June 2017; at least 1 year follow-up). The overall secondary arthrodesis rates did not change over the time period of 9 years while the 2-point distraction method was implemented gradually at the study site. No primary arthrodesis was performed. Elective hardware removals were performed in 34.9% of all patients due to subjective irritation of the screws, at patient request, or under the consideration that a secondary subtalar arthrodesis could interfere with the arthrodesis. The sustentaculum screw was left in situ in almost 90% of the patients. An overview of the performed implant removals and secondary subtalar fusions is presented in Table 4.

**Table 4:** Outline of implant removals performed, splitting regarding necessary and elective implant removal. A low obligatory following operation indication of 4.2% could be observed. Next, the rate of secondary subtalar fusions occurred with an overall incidence of 4.7%, which represents an arthrodesis rate comparable to the existing corpus of literature data

Year	Necessary implant removal		Elective implant removal		Secondary Subtalar Arthrodesis	
	N	% per year	N	% per year	N	% per year
2007	1	5.3%	12	63.2%	1	5.3%
2008	3	13.6%	5	22.7%	2	9.1%
2009	1	3.3%	9	30.0%	1	3.3%
2010	2	8.0%	14	56.0%	1	4.0%
2011	1	4.0%	9	36.0%	0	0.0%
2012	1	4.3%	7	30.4%	2	8.7%
2013	0	0.0%	8	36.4%	0	0.0%
2014	0	0.0%	9	33.3%	3	11.1%
2015	0	0.0%	1	5.3%	0	0.0%
Total	9	4.2%	74	34.9%	10	4.7%

The rate of unplanned revision surgeries between 2007 and 2010 was higher (7.3%) than that in the 2011–2015 period (1.7%). The rate of elective hardware removal between 2007 and 2010 was 41.7% compared to 29.3% between 2011 and 2015. The authors hypothesize, that these numbers can be attributed to the increasing experience with the minimally invasive technique and the modification of screw positioning.

## Discussion

We have modified and standardized the technique of minimally invasive operative treatment of intra-articular calcaneal fractures using the 2-point distractor through changes in positioning of the patient, intraoperative radiological viewing, screw placement adjustments, and postoperative care. We believe that this technique should be preferred over ORIF. The main advantage of this technique is the reduced rate of wound complications (2.7%) compared to ORIF (16.7%) in our study population (*p* < 0.01). Another important benefit is the possibility of performing the procedure immediately without waiting for consolidation of the soft tissue. This primarily affects the patient since an immediate operation reduces the pressure on the soft tissue and consequently reduces the level of pain, and it is also of socioeconomic interest as the duration of hospitalization is reduced due to less post-operative swelling. Last but not least, the overall arthrodesis rate of 4.7% at the Traumacenter Linz is comparable to the literature, although we treat all types of fracture morphologies via minimally invasive means, regardless of the amount of comminution. Also, if necessary, a secondary arthrodesis is technically easier to perform after minimally invasive procedures.

Many studies have been published concerning the optimal method of treating intra-articular calcaneal fractures [1–3, 6, 8, 10–14]. Most of them lacked a representative number of patients, and therefore, a general consensus is still undetermined [1, 2, 13].

At the Traumacenter Linz, the demographic analysis revealed relatively young patients (mean 43.3 years) which reflects the high socioeconomic influence of this fracture occurrence. Also, there is a male predominance of 3.8:1 in the study cohort. Causes of injury were high-energy trauma in the majority of patients. In accordance with the international literature, falls from heights are the most likely causes of injuries [15]. Contrary, Alexandridis et al. [16] and Bohl et al. [17] reported a lower incidence of falls, but a higher rate of traffic accidents (49%).

Open reduction and internal fixation (ORIF) has been the preferred therapy for intra-articular fractures in recent decades [1, 2]. The generally accepted approach for visualization of the fracture site is the extended L-shaped lateral approach, which is considered the gold standard [1]. Independent of the approach in ORIF, a consolidation of the soft tissues is recommended [2, 5, 18]. However, it can take 2–4 weeks before the so-called “wrinkle-sign” occurs and swelling decreases [2, 5]. Al-Mudhaffar et al. reported an increased incidence of wound healing problems when the operative procedures were performed within the first week post-injury in an open setting [18]. Rammelt et al. concluded that an operative intervention after 2 weeks also increases the complication rate, which could be explained by increasing fracture consolidation prior to surgery and a resulting need for higher force with reduction [5]. In our proposed technique, the surgery is intended to be performed within the first 3 days after injury. Even in cases with edematous tissue, our method did not lead to an increase in wound healing disturbances. The earlier the surgery was performed, the easier the mobilization of the fragments became.

To overcome problems with wound complications, wound infections, and skin necrosis specifically, many minimally invasive methods to reduce and fix calcaneal fractures have been proposed [14]. At the Traumacenter Linz, this rate was 2.7% using the 2-point distraction method. We believe this is not only a consequence of the operative technique, but also a result of early surgical intervention with hematoma evacuation, reduction, and stabilization thereby leading to a decrease in internal pressure.

In a meta-analysis, Fan et al. compared the clinical results after minimally invasive techniques to those after ORIF. The study reported a lower soft tissue complication rate, and reduced duration of the operative procedure itself. Also, functional results were almost equivalent for the two groups [12].

The minimally invasive technique has become a standardized procedure at the Traumacenter Linz, and about 86% of all operatively treated fractures have been utilized in the 2-point distractor technique.

Comparisons between the different methods of minimally invasive techniques and ORIF methods are difficult due to a lack of standardized measures, different techniques, and a low number of patients [11, 14, 19, 20].

Wallin et al. published a systematic review on the clinical results after minimally invasive techniques used to treat calcaneal fractures. The functional results after Sanders type II-IV compared with ORIF were promising, although most of the studies had low levels of evidence. Soft tissue complications and duration of the procedure were lower in the minimally invasive group. They did not discuss whether minimally invasive techniques or ORIF led to better anatomic reductions and functional results [14].

In a randomized controlled study, Kumar et al. found a lower rate of wound healing problems, and better functional outcomes in the minimally invasive group compared with those after ORIF. The authors postulated, that better functional outcomes can be explained by a lower wound complication rate and a better anatomical reconstruction [21]. In terms of anatomic reconstruction, ORIF is still considered the goldstandard for intra-articular fractures [1, 2, 8, 12].

In 2007, Schepers et al. presented their results on minimally invasive methods with a follow-up of 3 years. Functional results after minimally invasive techniques were lower compared to those reported after ORIF. The infection and wound complication rates were similar to those of ORIF. Subtalar joint motion could be restored to nearly 70% compared to the uninjured side. The secondary arthrodesis rate was higher than in Buckley’s study from 2002 [22, 23].

The most crucial factor for gaining a satisfactory result, according to Veltman, is the absence of complications [1]. Also, other authors concluded that the best results were achieved when both, the operative procedure, and aftercare were complication-free [2, 3]. Patients with comorbidities such as vascular diseases, diabetes, and nicotine abuse are more prone to these perioperative complications [2]. Also, patients over the age of 60 years are more likely to be affected by postoperative complications and subtalar arthritis, although this may be linked to the higher rate of comorbidities in elderly [2].

The published rate of arthrodesis is between 0 and 15% after minimally invasive surgery, 0–12% after ORIF, and 3.8–17% after conservative means [22–31]. The subtalar arthrodesis rate of 0% described by Park et al. was based on a relatively low number of patients and only 1 year follow-up [28]. In extremely complex intra-articular fractures, a primary subtalar arthrodesis is deemed the method of choice to achieve satisfactory results in the given situation [2].

The secondary subtalar osteoarthritis rate requiring arthrodesis after minimally invasive techniques was 4.7%. Between 2007 and 2015, this arthrodesis rate was almost constant. No primary subtalar arthrodesis was performed in our group of patients. The secondary arthrodesis rate of 4.7% after operative intervention in our study is comparable to the current body of literature. In a study published by Buckley et al., 37 of 218 (16.9%) patients required an arthrodesis, and 7 of 206 operatively treated patients (3.4%) required a secondary operative intervention [22]. More prospective randomized studies with longer follow-up periods are required to compare the results of minimally invasive techniques with ORIF and conservative treatment.

After conservatively treated calcaneal fractures, a 6-fold higher likelihood of arthrodesis has been published compared to primarily surgically treated patients. Furthermore, patients with Sanders Type IV, and patients with a Boehler angle of 0 degrees had a notably increased risk of secondary subtalar arthrodesis [32]. In general, operative reconstruction of calcaneal fractures provides a better tissue situation in cases which require a secondary subtalar arthrodesis thereby also leading to better long-term results [8].

Previous literature reported, that functional results after minimally invasive treatment are equivalent to those of ORIF [33, 34]. Based on these functional results, together with lower complication rates, two recent studies have argued that minimally invasive techniques are considered superior to open techniques in the treatment of intra-articular fractures [12, 14].

### Limitations

This study has certain limitations and weaknesses that must be considered.

First and foremost, no clinical evaluations or functional scores were assessed in the study cohort. This consequently limits comparability among patient groups. Therefore, this study was entirely focused on the rate of wound complications, the need for secondary arthrodesis, and relevant characteristics such as demographic data.

Future comparative studies are needed to verify the safety of operative procedures for calcaneus fractures. Whether this new technique will result in satisfactory long-term outcomes, or can prevent post-traumatic osteoarthritis should be determined in future prospective studies.

## Conclusions

We believe that minimally invasive procedures for the treatment of intra-articular calcaneus fractures can provide several benefits. These techniques lead to satisfactory results when the whole process from clinical and radiological examination, to indication, positioning of the patient, intra-operative X-ray views, reduction techniques, stabilization methods, and aftercare treatments are performed in a standardized fashion. The main advantage of our proposed technique is the low rate of wound complications compared to ORIF.

## Supplementary Information

**Additional file 1.** Supplement Technique.

## Data Availability

The datasets used and analyzed during the current study are available from the corresponding author at a reasonable request.

## Abbreviations

ORIF: Open reduction and internal fixation
K-Wires: Kirschner-wires
MIT: Minimal invasive technique
AUVA: Austrian Social Insurance for Occupational Risks

## References

[1] Veltman ES, Doornberg JN, Stufkens SA, Luitse JS, van den Bekerom MP (2013). Long-term outcomes of 1,730 calcaneal fractures: systematic review of the literature. J Foot Ankle Surg.

[2] Sharr PJ, Mangupli MM, Winson IG, Buckley RE (2016). Current management options for displaced intra-articular calcaneal fractures: non-operative, ORIF, minimally invasive reduction and fixation or primary ORIF and subtalar arthrodesis. A contemporary review. Foot Ankle Surg.

[3] Gougoulias N, Khanna A, McBride DJ, Maffulli N (2009). Management of calcaneal fractures: systematic review of randomized trials. Br Med Bull.

[4] Rammelt S, Gavlik JM, Zwipp H (2001). Historical and current treatment of calcaneal fractures. J Bone Joint Surg Am.

[5] Rammelt S, Zwipp H (2004). Calcaneus fractures: facts, controversies and recent developments. Injury.

[6] Meena S, Gangary SK, Sharma P (2016). Review article : operative versus non- operative treatment for displaced intra- articular calcaneal fracture : a meta-analysis of randomised controlled trials. J Orthop Surg.

[7] Ibrahim T, Rowsell M, Rennie W, Brown AR, Taylor GJS, Gregg PJ (2007). Displaced intra-articular calcaneal fractures: 15-year follow-up of a randomised controlled trial of conservative versus operative treatment. Injury.

[8] Guerado E, Bertrand ML, Cano JR (2012). Management of calcaneal fractures: what have we learnt over the years?. Injury.

[9] Radnay CS, Clare MP, Sanders RW (2010). Subtalar fusion after displaced intra-articular calcaneal fractures: does initial operative treatment matter?. J Bone Jt Surg Am.

[10] Epstein N, Chandran S, Chou L (2012). Current concepts review: intra-articular fractures of the calcaneus. Foot Ankle Int.

[11] Schepers T, Patka P (2009). Treatment of displaced intra-articular calcaneal fractures by ligamentotaxis: current concepts’ review. Arch Orthop Trauma Surg.

[12] Fan B, Zhou X, Wei Z, Ren Y, Lin W, Hao Y (2016). Cannulated screw fixation and plate fixation for displaced intra-articular calcaneus fracture: a meta-analysis of randomized controlled trials. Int J Surg.

[13] Jiang N, Lin QR, Diao XC, Wu L, Yu B (2012). Surgical versus nonsurgical treatment of displaced intra-articular calcaneal fracture: a meta-analysis of current evidence base. Int Orthop.

[14] Wallin KJ, Cozzetto D, Russell L, Hallare DA, Lee DK (2014). Evidence-based rationale for percutaneous fixation technique of displaced intra-articular calcaneal fractures: a systematic review of clinical outcomes. J Foot Ankle Surg.

[15] Mitchell MJ, McKinley JC, Robinson CM (2009). The epidemiology of calcaneal fractures. Foot (Edinb).

[16] Alexandridis G, Gunning AC, van Olden GDJ, Verleisdonk EJMM, Segers MJM, Leenen LPH. A trauma system wide evaluation of the demographic, injury and fracture characteristics of patients with calcaneal fractures: a comparison of trauma level I and II centers. Clin Res Foot Ankle. 2017;5:237. 10.4172/2329-910X.1000237.

[17] Bohl DD, Nathaniel T, Samuel AM, Diaz-collado PJ, Stephen J, Basques BA (2016). A study of 14 516 patients in the American College of Surgeons National Trauma Data Bank. Foot Ankle Spec.

[18] Al-Mudhaffar M, Prasad CV, Mofidi A (2000). Wound complications following operative fixation of calcaneal fractures. Injury.

[19] Pelliccioni AAA, Bittar CK, Zabeu JLA (2012). Surgical treatment of intraarticular calcaneous fractures of sanders’ types II and III. Systematic review. Acta Ortop Bras.

[20] Bhattacharya R (2005). Sanders classification of fractures of the os calcis: an analysis of inter- and intra-observer variability. J Bone Jt Surg Br.

[21] Sampath Kumar V, Marimuthu K, Subramani S, Sharma V, Bera J, Kotwal P (2014). Prospective randomized trial comparing open reduction and internal fixation with minimally invasive reduction and percutaneous fixation in managing displaced intra-articular calcaneal fractures. Int Orthop.

[22] Buckley R, Tough S, Mccormack R, Pate G, Leighton R, Petrie D (2002). Operative compared with nonoperative treatment of displaced intra-articular calcaneal fractures. J Bone Jt Surg Am.

[23] Schepers T, Schipper IB, Vogels LMM, Ginai AZ, Mulder PGH, Heetveld MJ (2007). Percutaneous treatment of displaced intra-articular calcaneal fractures. J Orthop Sci.

[24] Stulik J, Stehlik J, Rysavy M, Wozniak A (2006). Minimally-invasive treatment of intra-articular fractures of the calcaneum. J Bone Jt Surg Br.

[25] Tomesen T, Biert J, Frolke JP (2011). Treatment of displaced intra-articular calcaneal fractures with closed reduction and percutaneous screw fixation. J Bone Joint Surg Am.

[26] De Vroome SW, Van Der Linden FM (2014). Cohort study on the percutaneous treatment of displaced intra-articular fractures of the calcaneus. Foot Ankle Int.

[27] Tantavisut S, Phisitkul P, Westerlind BO, Gao Y, Karam MD, Marsh JL (2017). Percutaneous reduction and screw fixation of displaced intra-articular fractures of the calcaneus. Foot Ankle Int.

[28] Park J, Che JH (2017). The sinus tarsi approach in displaced intra-articular calcaneal fractures. Arch Orthop Trauma Surg.

[29] Ebraheim NA, Elgafy H, Sabry FF, Freih M, Abou-Chakra IS (2000). Sinus tarsi approach with trans-articular fixation for displaced intra-articular fractures of the calcaneus. Foot Ankle Int / Am Orthop Foot Ankle Soc [and] Swiss Foot Ankle Soc.

[30] Griffin D, Parsons N, Shaw E, Kulikov Y, Hutchinson C, Thorogood M (2014). Operative versus non-operative treatment for closed, displaced, intra-articular fractures of the calcaneus: randomised controlled trial. BMJ.

[31] Agren P, Wretenberg P, Sayed-Noor AS (2013). Operative versus nonoperative treatment of displaced intra-articular calcaneal fractures: a prospective, randomized, controlled multicenter trial. J Bone Jt Surg.

[32] Csizy M, Buckley R, Tough S, Leighton R, Smith J, McCormack R (2003). Displaced intra-articular calcaneal fractures: variables predicting late subtalar fusion. J Orthop Trauma.

[33] Van Hoeve S, Poeze M (2016). Outcome of minimally invasive open and percutaneous techniques for repair of calcaneal fractures: a systematic review. J Foot Ankle Surg.

[34] Giannini S, Cadossi M, Mosca M, Tedesco G, Sambri A, Terrando S (2016). Minimally-invasive treatment of calcaneal fractures: a review of the literature and our experience. Injury.

