# Supplementary material for: Minimally invasive surgery for intra-articular calcaneus fractures: a 9-year, single-center, retrospective study of a standardized technique using a 2-point distractor
Source: BMC Musculoskelet Disord. 2020 Nov 14;21:753. doi: 10.1186/s12891-020-03762-9 (PMC7666766; doi:10.1186/s12891-020-03762-9)
Supplement: Supplementary file 1 — Additional file 1. Supplement Technique. [file 12891_2020_3762_MOESM1_ESM.zip › Additional_file 1_Supplement_TechniqueR2.docx]

***Supplement Technique***

**"Minimally invasive surgery for intra-articular calcaneus fractures: A 9-year, single-center, retrospective study of a standardized technique using a 2-point distractor"**

**Reduction technique – selection of method and preparation**

We distinguished 4 groups of fracture types. The Essex - Lopresti classification system was used because of its simple characteristics and good relationship with therapeutic decision-making, reduction technique, and clinical outcome. We treated all types of fractures via minimally invasive procedures and worked to continuously improve the treatment concepts, instruments, and implants.

These 4 groups are discussed:

1. Essex-Lopresti tongue-type fractures
2. Essex-Lopresti depression-type fractures
3. Highly comminuted fractures in depression-type forms
4. Atypical fractures with uncommon fracture lines, mainly following direct trauma.

**Tongue-type fractures**

Tongue-type fractures have a large dorsal segment of the tuber calcaneus connected to a calcaneal subtalar joint fragment. Shortening and varus malalignment of the foot is minimal. The Westhues maneuver allows for good reduction with this type of fracture. ^7^

**Depression-type fractures**


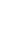
The characteristic fracture line runs between the tuber calcanei and the central calcaneal body. In simple fractures, an isolated lateral joint fragment and usually a break-out of the lateral wall are present. In more severe cases, there are multiple central fragments and a broken sustentaculum tali. Shortening, varus malalignment, depression of the subtalar joint, tilting of the talus, broadening, and a broken lateral wall, often occur with impingement of the upper ankle joint and/or fracture of the lateral ankle. The extension technique with a 2-point distractor is recommended. ^20^

**Highly comminuted fractures**

Comminuted fractures have increasing fragmentation and dislocation due to greater force, but similar trauma mechanism compared to depression type fractures

These kinds of fractures provide the main indication for correcting the axis, length, broadening and height, and fixation of the main fragments via minimally invasive techniques using the 2-point distractor in the early stages after injury. Therefore, surgery without delay is suggested.

**Atypical** **fractures**

Fractures with an atypical mechanism of injury and an uncharacteristic progression of the fracture lines most often result from direct trauma. In most cases, standard reduction techniques with screw fixations are performed. Depending on the fracture pattern, alternative reduction and fixation techniques should be considered.

**Contraindications**

At our center, all fracture types, from simple to comminuted, as well as open injuries are treated via minimally invasive methods as described in the following sections. Contraindications are rare. Because of the reduced operative risks, our protocol can be performed in older patients, smokers, and patients with comorbidities.

**Anesthesia**

Surgery was typically performed under regional anesthesia. Tourniquets were not usually applied, and postoperative pain catheters were often useful.

**Positioning - imaging**

A standardized, stable position of the patient with a full approach to the foot and a properly adjusted image intensifier were pre-conditions for success with this operative technique. We used the same positioning for all fracture’s types. An iso-centric image intensifier with optional intraoperative computed tomography (CT) imaging was advantageous. Without this, the intraoperative Brodén views could only be applied with additional height adjustments.

Under this protocol, the patients were placed in lateral decubitus position with the injured foot on a leg-holder in the upper and strictly horizontal position (Figure 1). Free access to the entire foot was essential for proper mounting of the distraction device. The C-arm was placed so that it could move 360 degrees around the foot without changing the position and/or height of the image intensifier itself. In this protocol, it is highly recommended to prepare, standardize, document, and train the operative staff and radiology technicians, collectively, on the positioning and usage of the equipment prior to surgery.

In the minimally invasive approach, the fracture segments are not directly visualized and therefore optimal intraoperative imaging is necessary for success. It is essential that the surgeons are entirely familiar with the imaging technique to accurately instruct the radiology technicians intraoperatively. Imaging uncertainties during surgery should be avoided.

We utilized 3 standardized intraoperative views: lateral, Brodén, and axial.

The image intensifier was positioned exactly in-line with the longitudinal axis of the calcaneus. The foot was positioned in a strictly horizontal position, and all 3 intraoperative views were set and eventually marked. Afterwards, the image intensifier wheels were locked in position, and only the C-arm remained mobile. This minimized the amount of time required to move the C-arm in and out of the operative field and ensured consistent views. Only 3 different instructions for intraoperative imaging were needed for radiological assistance. Surgery was started only after appropriate positioning of the imaging unit was set.

**Lateral view**

Exact orthogonal adjustment of the talus is important. The calcaneus should be disregarded when adjusting the image intensifier (because it is broken and misshapen). Lateral ligament instability due to subluxation can impede the adjustment of the image intensifier.

**Brodén view**

This projection was used to evaluate subtalar joint and posterior facet congruency. After the previous adjustment, the C-arm was angled 45 degrees cephalad, while using an iso-centric image intensifier. Because of the curvature of the talus, a ´rotated’ fracture fragment could be seen as normal in the Brodén view at different degrees. To avoid missing rotated or dislocated fragments, the posterior facet should always be checked at a tube angle of 35 and 55 degrees intraoperatively.

**Axial view**

The C-arm was adjusted horizontally. In manual dorsal flexion of the foot evaluation of the axis of the calcaneus was possible. This view was essential for exact positioning of the pins.

**Reduction techniques**

***Westhues reduction method***

The Westhues technique was typically performed with tongue-type calcaneus fractures. For the reduction, we used a Schanz screw from the dorsal direction.

Fixation was performed using 4 mm sustentaculum screws and 6.5, or preferably, 7.3 mm fully-threaded static screws (Figure 2).

***Reduction using an extension device***

We found that minimally invasive techniques using the 2-point distractor were effective for the treatment of joint-depression-type and comminuted fractures. We used the distraction device by Implantate Technologie System GmbH (ITS. GmbH - Lassnitzhöhe, Styria, Austria) and described by Fröhlich et al. in 1999 (Figure 3). ^12^

The procedure started with the insertion of two 3.2 mm pins in the talus and calcaneus. The first pin was inserted in the anterior process of the talus, exactly in the frontal and axial planes, while alignment was assessed via the lateral view (Figure 4 – red spot).

The second pin was inserted into the distal plantar region of the calcaneal tuber, perpendicular to the varus-tilted length axis of the tuber fragment via the axial view (Figure 4 – blue spot). With the varus malalignment of the tuber fragment, a significant convergence of the pins on the medial side must be obvious (Figure 5), otherwise, correction of the varus using the 2-point-distractor with fixed axes is not possible, and the reduction will fail.

Next, the varus malalignment was manually corrected. The distractor was mounted on the medial side first, then on the lateral side. The pins were then bent over and pinched off. Extension was applied alternately while using the lateral and axial views to reconstruct the calcaneal length and create space to reduce the central joint fragments (Figure 6). The extension device remained until the end of the operation, and the foot was stabilized in preparation for the screw fixation. Besides correcting the varus and length, the longitudinal arch was also raised with the help of ligamentotaxis (especially the plantar fascia) - thus correcting Boehler’s angle.

***Reduction and stabilization of the central joint fragments***

The most common approach utilized a stab incision on the lateral side (Figure 7). Reduction was performed using a raspatory or elevator directly, or through the fractured lateral wall. Additionally, we evacuated any fracture hematomas. We also used some different techniques with the elevator aimed from the dorso-lateral direction, or from the plantar aspect using a plunger.

Stabilization of the fragment was performed with 1 or 2 sustentaculum screws. The sustentaculum usually remains intact because of the typical fracture mechanism, the powerful talocalcaneal ligaments, and the strong trabecular structure of the sustentaculum tali itself. The sustentaculum screw was placed from the lateral-dorsal to the medial-ventral direction. The target was the sustentaculum tali directly below the center of the medial malleolus. Due to the complex anatomical shape of the sustentaculum and difficult visualization, correct placement of the sustentaculum screw was challenging. Temporary K-wires were used to prepare for the insertion, which was performed under fluoroscopic guidance in all 3 views. We usually used 4.0 mm lag screws. With correct positioning, good compression was observed in the Brodén view.

When necessary, reduction and fixation of the anterior process, the tuber fragment, or other fragments were performed.

***Stabilization of length, height and axes***

We generally used two 7.3 mm cannulated, fully-threaded screws for internal fixation In the literature, many different placement techniques are described. ^12,31^ We recommend an entry point above the insertion of the Achilles tendon, superior to the tuber pin, and angulated in a cranial-to-plantar direction. The advantage is less local soft tissue irritation, good fixation of the screw heads because of stronger and thicker distal cortices, and support of central joint fragments (like “roof beams“) (Figure 8). This becomes even more important when bone defects are present after a reduction due to compression of cancellous bone. Autologous bone or bone substitute grafts are not necessary in our opinion, and recent literature shows the same. Heads of screws should be placed subcortically. Alternatively, headless screws can be used.

***Postoperative treatment***

Postoperatively, we did not drain the wound. Functional treatment after fixation through screw osteosynthesis was usually possible and should be intended. Sometimes we used plaster cast fixation until wound healing was complete. Early supervised physical therapy should be prescribed. Passive movement of the joint within a painless range of motion increases mobility, limits postoperative decalcification of the bones, and positively affects cartilage regeneration and healing. In the past, non-weightbearing was prescribed for 12 weeks; however, we have seen sufficient bone healing after 6 weeks after stable fixations. Therefore, we have started prescribing increasing levels of weightbearing activity after 6 weeks. Removal of the screws is not necessary, but sometimes we have observed slightly painful scarring on the heel, at which point we remove the 7.3 mm screws. These can be removed under local or regional anesthesia.

**Figure Legends**

**Figure 1:** Lateral decubitus position with leg in the leg-holder

**Figure 2:** Westhues reduction method utilizing a Schanz screw as a joystick

**Figure 3:** Froehlich extension device

**Figure 4:** Insertion points of 3-mm pins

**Figure 5:** Convergence of 3.2-mm pins on the medial side as result of varus malalignment of the tuber fragment

**Figure 6:** Extension device is mounted on the medial and lateral sides, and a distraction force is applied

**Figure 7:** Stab incision on the lateral side to facilitate reduction of the posterolateral joint fragment using a raspatory under fluoroscopic guidance

**Figure 8:** Static fixation with fully-threaded screws to retain the length and axis of the calcaneus with two sustentaculum screws from dorsolateral to anteromedial
