# Supplementary figures and images for: Minimally invasive surgery for intra-articular calcaneus fractures: a 9-year, single-center, retrospective study of a standardized technique using a 2-point distractor
Source: BMC Musculoskelet Disord. 2020 Nov 14;21:753. doi: 10.1186/s12891-020-03762-9 (PMC7666766; doi:10.1186/s12891-020-03762-9)

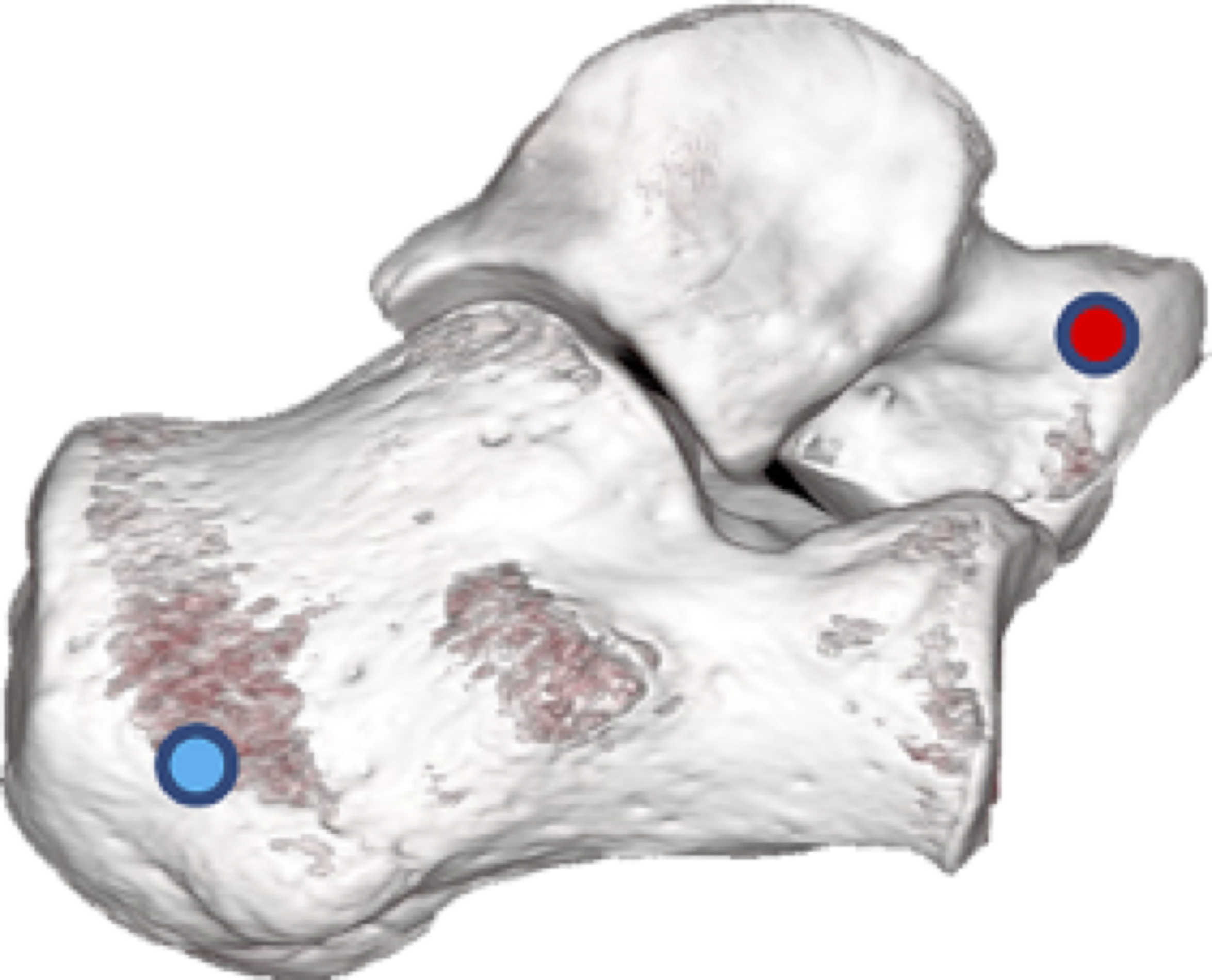

Supplement: Supplementary file 1 — Additional file 1. Supplement Technique. [file 12891_2020_3762_MOESM1_ESM.zip › Figure 4 SupplementR2.tiff]

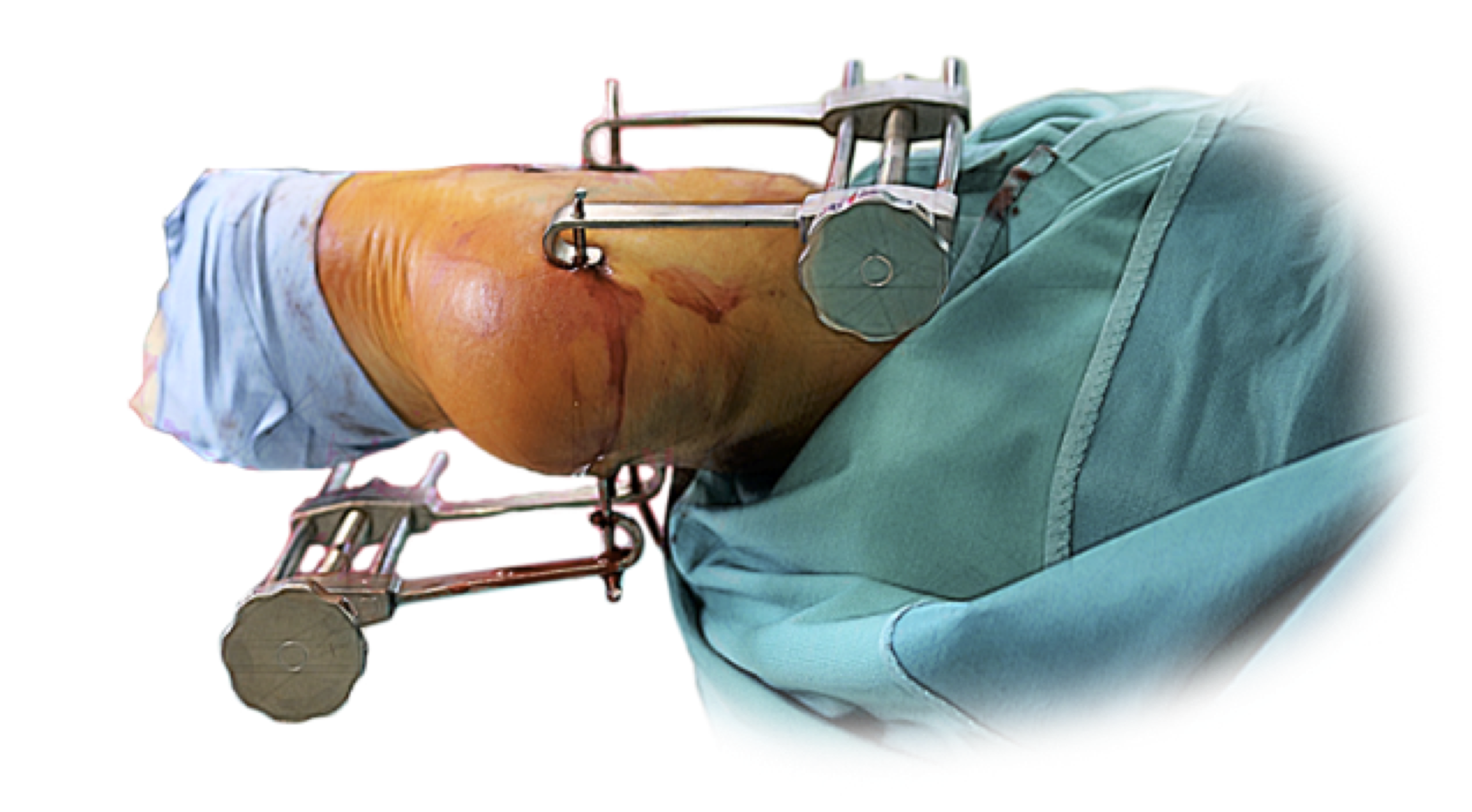

Supplement: Supplementary file 1 — Additional file 1. Supplement Technique. [file 12891_2020_3762_MOESM1_ESM.zip › Figure 6 SupplementR2.tiff]

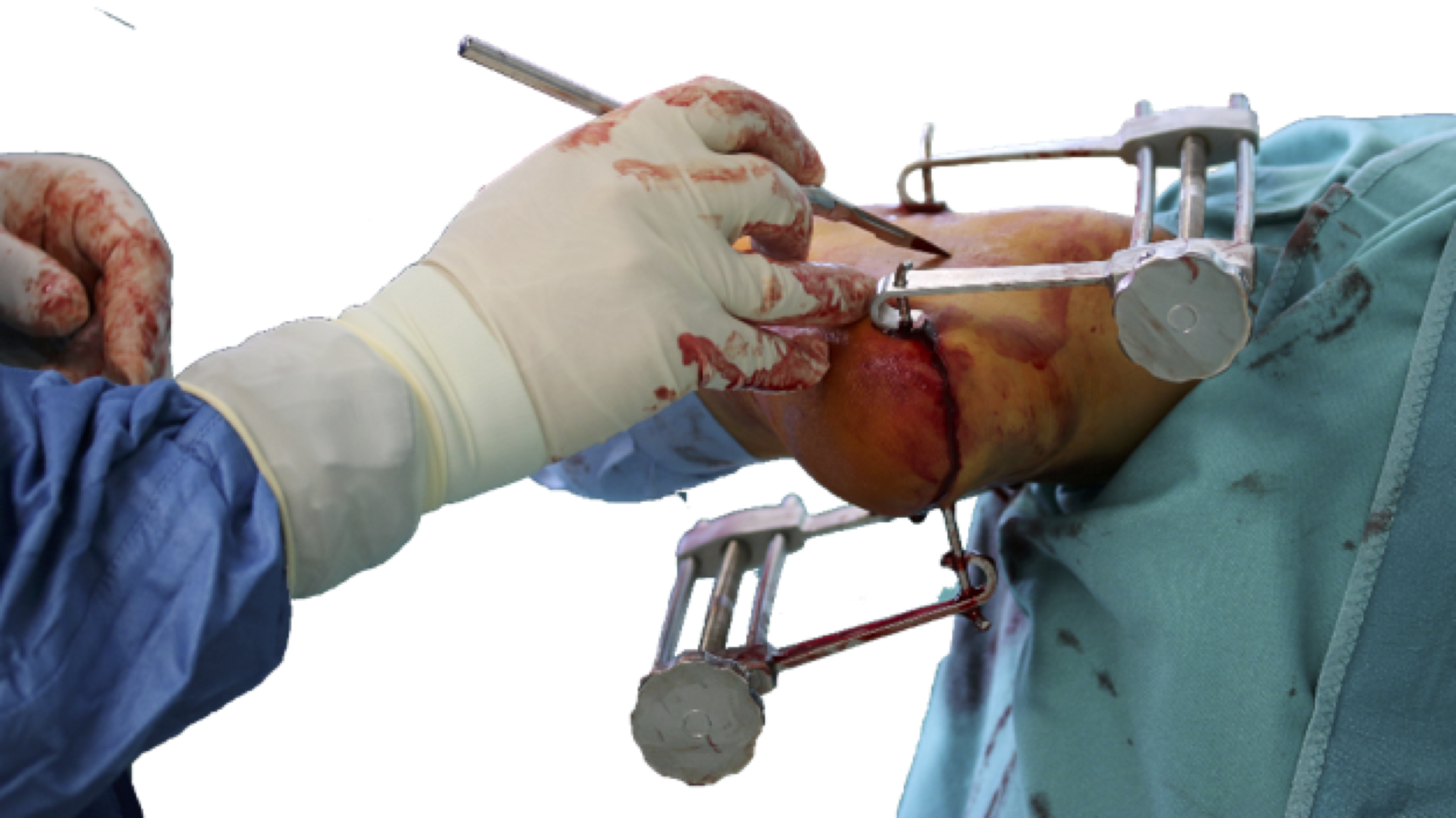

Supplement: Supplementary file 1 — Additional file 1. Supplement Technique. [file 12891_2020_3762_MOESM1_ESM.zip › Figure 7 SupplementR2.tiff]

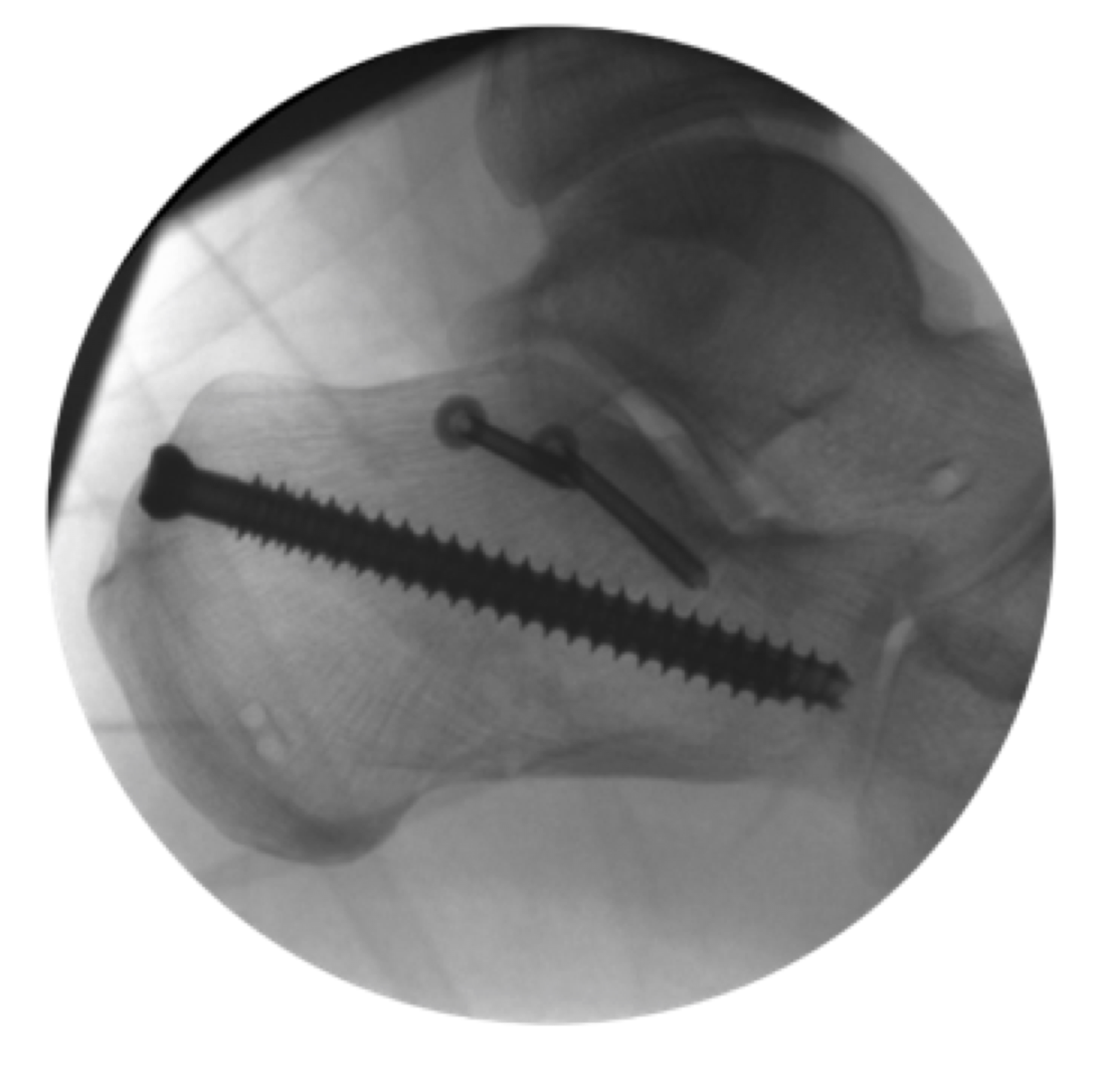

Supplement: Supplementary file 1 — Additional file 1. Supplement Technique. [file 12891_2020_3762_MOESM1_ESM.zip › Figure 8 SupplementR2.tiff]
